# Dataset of user evaluations of prototypicality, aesthetics, usability and trustworthiness of homepages of banking, e-commerce and university websites

**DOI:** 10.1016/j.dib.2023.109976

**Published:** 2023-12-18

**Authors:** Aliaksei Miniukovich, Kathrin Figl

**Affiliations:** a Department of Information Systems Production and Logistics Management, University of Innsbruck, Universitätsstraße 15, A-6020 Innsbruck; b Department of Design, Norwegian University of Science and Technology, Raufossvegen 40, 2821 Gjøvik

**Keywords:** Web design, First impression, User preference, Crowdsourcing

## Abstract

The dataset contains full-page screenshots of homepages of commercial banking (*N* = 1033), online shopping (*N* = 1064), and university (*N* = 1059) websites, as well as the raw and aggregated user ratings of webpage design prototypicality, visual aesthetics, perceived usability and trustworthiness, and user demographic information. Design prototypicality was measured with three items, including typicality, exemplar goodness, and family resemblance, whereas the other design dimensions were measured with a single item each. Amazon Mechanical Turk crowdworkers (*N* = 3319 rating sessions) provided their demographic data and rated the homepages online. The demographic data have been anonymized, with generated unique participant IDs replacing MTurk crowdworker IDs. The screenshots are identified with generated IDs to provide partial anonymization for the websites, limiting their potential misuse outside design-related or user experience-related academic research. The raw rating data contain all collected ratings, whereas the aggregated data contain the per-webpage, per-dimension ratings derived solely from the ratings of study-compliant crowdworkers. The non-compliance among crowdworkers was detected based on several indicators, including rate-rerate consistency, seen-unseen webpage recognition, free-form feedback analyses, demographic data analyses, and other indicators. Future research could utilize the dataset either in user studies that require full-page webpages as stimuli, e.g., studies on the determinants of first impression, user preference, and user experience, or in computational research on web design, including computational aesthetics, as this type of research requires a large number of user-rated webpages, which this dataset provides.

Specifications Table

**Table:** 

Subject	Computer Science: Human-Computer Interaction
Specific subject area	User first impression of webpage design quality
Data format	Raw; Filtered and Aggregated
Type of data	Table Image
Data collection	An online study, primarily involving US MTurk crowdworkers, evaluated the webpages on their prototypicality, visual aesthetics, perceived usability, and trustworthiness. The screenshots of the webpages were at full-width (1440px) and scrolled down automatically, with a 7-point rating scale shown at the bottom after scrolling. Demographic data were collected prior to the rating trials, and seen-unseen webpage recognition data after the trials. Data from non-compliant crowdworkers (identified by a failed recognition test or low rate-rerate consistency) were removed before the ratings were averaged. Individual ratings were within-participant standardized, and then arithmetic averages were calculated for per-dimension per-webpage scores.
Data source location	University of Innsbruck Department of Information Systems Production and Logistics Management Universitätsstraße 15 A-6020 Innsbruck
Data accessibility	Repository name: Web Design Prototypicality Data Data identification number: https://doi.org/10.7910/DVN/9FKSQI https://doi.org/10.7910/DVN/XOI0HI https://doi.org/10.7910/DVN/Z7KLIH Direct URL to data: https://dataverse.harvard.edu/dataverse/webdesignprototypicality/ Instructions for accessing these data: Follow the link and access the nested three datasets - they are available for download.
Related research article	A. Miniukovich, K. Figl. The Effect of Prototypicality on Webpage Aesthetics, Usability, and Trustworthiness, International Journal of Human-Computer Studies (2023) https://doi.org/10.1016/j.ijhcs.2023.103103

## 1 Value of the Data

• The data [1] describe the user perception of several visual aspects of webpage design, and can be used in future human-computer interaction (HCI) research and practice. The designs belong to three web domains - commercial banking, e-commerce, and tertiary education - allowing studying the differences between the domains. Unlike in previous datasets with a similar purpose (c.f., [2]), the webpages were captured as full-page screenshots instead of screenshots of top-screen webpage parts. The dataset is large enough for machine-learning applications.
• HCI researchers and practitioners could benefit from the data, including researchers studying the user perception of graphical user interfaces (GUIs), and webpages in particular; researchers developing computational models of GUI visual aesthetics and prototypicality; and practitioners developing systems for automatic webpage design search, comparison, and analysis.
• Other researchers could utilize the webpage screenshots as stimuli in their user studies of design perception, first impression, and preference. Since the webpages are already pre-rated on visual aesthetics, perceived usability, trustworthiness, and prototypicality, the researchers could skip collecting these data, and either focus on evaluating complementary dimensions (e.g., perceived design orderliness or information quality) to compare against the already-rated dimensions, or use the already-rated dimensions as potential confounding factors to control for. Researchers could also use the webpages and ratings for training computational models of GUI quality, including GUI visual aesthetics.

## 2 Data Description

The data reside in three datasets, each with an identical structure but describing a different web domain: commercial banking, fashion and homeware e-commerce, and tertiary education. Each dataset contains two types of data, images (JPEG) and data tables. The images are 1000+ full-page screenshots of website homepages for each web domain, with the screenshots structured in groups of ∼100 in individual sub-folders (because the data repository could not facilitate uploading all screenshots at once). The names of screenshot image files combine a letter (“b” for commercial banks, “f” for fashion e-commerce, “h” for homeware e-commerce, and “u” for universities) and a unique identifier number (no special meaning; starting from one and incrementing the number by one for the next screenshot). Three screenshots per dataset were used for training and have a “t” (stands for “training”) after the letter in their name.

The data tables, three for each domain, are all tab-separated and saved as text in .txt files. The “*ratings.raw.[domain].txt*” files contain the raw ratings of all participants who viewed and rated the webpages, including the ratings of suspected non-compliant participants, whose ratings were subsequently excluded from aggregation. Each row is an individual rating (Table 1).

**Table 1:** The columns of data tables with raw rating data. Each row in the data tables is a rating.

Column name	Data type	Description
stimulusId^1^	String	The name of the screenshot image, to which a rating corresponds
isDuplicate	Boolean	Shows “true” if a webpage was rated the second time by a participant. The rate-rerate ratings were used for quality control (a compliant participant was presumed to rate the same stimuli relatively consistently).
rating	Integer	A rating refers to the evaluation provided by a participant for a specific dimension of a webpage, range: [-3,3]
isTraining	Boolean	Shows “true" for the webpages rated for training in the beginning of each session
dimension	Factor	The ID of the dimension that a rating corresponds to, with TYP for typicality, AVG for family resemblance, EXMPL for exemplar goodness, AE for visual aesthetics, US for usability, and TRU for trustworthiness
sessionId	String	A unique ID for a data collection session.

1 The stimulus IDs are neither derived from nor related to the URLs of the webpages. If linking the IDs to the URLs is needed, the corresponding author can provide such information upon request for academic research purposes.

The “*ratings.avg.[domain].txt*” files contain filtered and aggregated data. Each row describes one stimulus, with the column “stimulusId” identifying the stimulus and the other six columns (TYP, AVG, EXMPL, AE, US, TRU) being the ratings for the stimulus on each of the six dimensions. The ratings of non-compliant participants were filtered out before the aggregation. The aggregation included three steps: first, averaging (using the arithmetic mean) the ratings of the twice-rated webpages for each participant; next, the ratings were standardized within each participant (cf., [3]); and finally, averaging (using the arithmetic mean) all ratings across participants for each webpage for each dimension.

The “*demographics.[domain].txt*” files contain demographic data and recognition test data, Table 2. Each row describes a session, not a participant, since non-compliant participants were allowed to repeat the study and had to report their demographic data again as an additional data quality control (e.g., by checking for mismatched or misreported demographic details).

**Table 2:** The columns of demographic data tables. Each row is a data collection session.

Column Name	Data Type	Description
sessionId	String	A unique ID for a data collection session. It links demographic data with rating data.
uid	String	A unique Participant ID
Gender	Factor	Self-reported participant gender. Options: {Female, Male, Other, RefuseToAnswer}
Education	Factor	Self-reported highest level of education obtained. Levels: {Middle school or less - Doctorate degree}
Age	Integer	Self-reported participant age (18 and above, data collection included adults)
Country	String	Self-reported participant country of origin
Occupation	Factor	Self-reported participant occupation options: {Student, Training/Apprenticeship, Partially Employed, Fully Employed, Unable to Work, Self-Employed, Military, Retired, Homemaker, Out of work/Unemployed, Other}
Vision	Factor	Whether a participant self-reported being color-blind. Options: {Normal, ColorBlind}
EngFluency	Boolean	Whether a participant self-reported speaking English fluently
OtherLang	String	Other languages that a participant self-reported to browse the Web in
WebUse	Numeric	Number of hours per day a participant self-reported to browse the web (range: 0-24)
ShopFamiliarity; UnivFamiliarity; BankFamiliarity	Integer	A participant's self-rated familiarity with websites of one of three studied web domains (range: 1-7, anchors Low and High)
wscreen; hscreen	Integer	The width and height of the browser window used in the current study session
t2send	Integer	Number of milliseconds taken to fill out and submit the demographics questionnaire
ifVpn	Boolean	Whether a participant used a VPN
precision	Numeric	Recognition test performance, ratio of true positives (truly seen webpages among claimed seen webpages) to all positives (claimed seen webpages)
recall	Numeric	Recognition test performance, ratio of true positives (truly seen webpages among claimed seen webpages) to relevant items (truly seen webpages, always n = 10)
accuracy	Numeric	Recognition test performance, ratio of correctly detected seen (10 webpages) and unseen (also 10) webpages to the total number of webpages in a test (20 in total)
susCheat	Boolean	Whether the current session was detected to be for a non-compliant participant (untrustworthy data)

## 3 Experimental Design, Materials and Methods

### 3.1 Stimuli

The screenshots of website homepages were the stimuli. The websites belonged to one of three web domains: commercial banking, apparel and homeware ecommerce, and tertiary education. These domains were chosen for practical reasons: they are well-known to the average user (the user could evaluate them confidently and consistently); large (featuring many websites within them); not dominated by a single example (unlike, for example, the search domain, which is often dominated by google.com); visually diverse (unlike, for example, many news websites that often re-use the same visual template with only minor customizations); and relatively well-defined (with the semantic boundaries between domains being clear to an average participant). Homepages, rather than other webpages, were used because they appeared to be the most representative of the overall look-and-feel of the websites.

We collected the URLs of websites to screenshot from open sources, e.g., the lists of websites on Wikipedia or the US government's list of banking institutions, and by using a search engine. This process yielded several thousand potential URLs for both commercial banking and tertiary education, but fewer than 1500 URLs for e-commerce. From the thousands of URLs for banking (N_total_ = 7564) and education (N_total_ = 9455), 1500 were randomly sub-selected for both categories. Gathering as many URLs as practically possible and randomly sub-selecting from them was intended to minimize potential sampling biases. The sub-selected URLs were then manually reviewed and only kept if the webpages that they led to were homepages (and not, say, language-selection pages or stub pages), were not visibly broken, were not for a localized version of an already kept webpage (e.g., zalando.co.uk and zalando.com), were mostly in English, and were from the target web genre. Assuring the latter included, for example, filtering out the websites of central banks and wealth management funds from the commercial banking genre, websites without visible prices from the e-commerce genre, and overtly religious institutions from the tertiary education genre. The resulting samples included 1030 screenshots for commercial banking, 1056 for tertiary education, and 1058 for e-commerce (550 apparel, and 508 homeware).

A custom-developed extension for Firefox 88.0 captured the screenshots. Prior to screenshotting, the webpages were modified to ensure they looked as closely as possible to what the user would see in a browser. The modifications included force-loading images (else a browser might not load them until the user scrolls down past their position), freezing animations, removing one-time announcements (e.g., GDPR-related cookie notices), removing page-covering overlays (e.g., overlays soliciting user emails for newsletters), repositioning and fixing in-place scrolling-dependent elements (otherwise they would appear out of place on a full-page screenshot), moving menus and elements attached to the bottom of the screen down to the bottom of a page, and other necessary modifications. The screenshots were full-page height and width, which meant a varying length, but mostly identical width - 1440px, the most commonly used browser window width - across them, with a few screenshots being wider due to their corresponding webpages being wider.

### 3.2 Procedure

The data collection was conducted online, separately for each web genre, but with nearly identical procedures. Participants were recruited on the Amazon Mechanical Turk crowdsourcing platform and redirected to a custom-developed website. They then read the data collection description and conditions, and upon accepting them, were redirected to a demographics questionnaire. After the questionnaire, they rated 83 webpages. Among these, three were for training (these were always presented first and were the same for all participants), and twelve were duplicates (the same webpage was shown again, at least eight trials away from its initial presentation). Each rating trial included a gray mask for .75-1.25 seconds, a screenshot that was automatically scrolled down, and a scale below the screenshot to rate it on a design dimension (Table 3). The dimension was always the same for a participant. The automatic scrolling paused for three seconds on the top-screen webpage part, two seconds on the webpage part under it, and one second for all consecutive parts. To prevent participants from skipping to the rating section before viewing a webpage fully, the in-browser keyboard shortcuts for scrolling down were disabled. After the 83 trials, participants completed a recognition test. The test included ten seen and ten unseen webpages, shown as thumbnails (360 by 525 pixels), arranged in a grid. Participants needed to select only the webpages they had seen. After the test, participants could leave optional free-form feedback as text. Participation was compensated with 2.1 USD.

**Table 3:** Measurement items. The scale was always 7-point, from -3 to 3. Webpage prototypicality was evaluated with three items. Aesthetics, usability, and trustworthiness were evaluated with one item each, similar to the existing practices for rating webpages in related prior studies [4,5].

Construct	Item	Text	Low Anchor	High Anchor	Related Measures
Prototypicality	Exemplar Goodness	This webpage is a representative example of homepages of university/online-shopping/commercial-bank websites.	Not at all	Very Much	3-item measure of perceived typicality [6]
	Family Resemblance	This webpage has many visual aspects in common with homepages of other university/online-shopping/commercial-bank websites.			
	Typicality	This webpage looks like a typical homepage of a university/online-shopping/commercial-bank website.			
Visual Aesthetics		This webpage looks	Ugly	Beautiful	VisAWI [7]; classical and expressive aesthetics [8]
Perceived Usability			Difficult to Use	Easy to Use	SUS [9]; 5-item usability metric [8]
Trustworthiness			Not Trustworthy	Trustworthy	3-dimensional metric of trustwor-thiness[10]

### 3.3 Participants

A total of 2822 MTurk crowdworker accounts participated in the data collection. Crowdworkers were recruited from several predominantly English-speaking countries, but the vast majority of them came from the USA. Nearly all participants self-reported speaking English fluently and having normal color vision. The participant sample was balanced and representative of the US average in terms of age, gender, occupation, and education level, but likely included participants with considerably more web browsing experience than the average person, as MTurk crowdwork is an online activity.

### 3.4 Data Quality

To counter the low quality of crowdsourcing data [11], we used several non-trivial indicators to detect non-compliant MTurk accounts and filter their data out from the per-webpage per-dimension averages to be used in eventual analyses (the “*ratings.avg.[domain].txt*” files). First, we looked at the consistency of ratings for the twice-rated webpages, since a compliant participant was expected to not be completely inconsistent with themselves when rating the same webpage on the same item within the same session. Second, we expected compliant participants to perform at least slightly better on the seen-unseen recognition test than random guessing. Third, we expected compliant participants not to use one of the few phrases that bot accounts use for free-form feedback. Finally, we used several simpler indicators to complement the non-trivial indicators, which included checking if an account tried to hide their IP address with a VPN, if demographic data were bot-like (e.g., non-compliant accounts seemed to default to the age of 25, possibly due to using scripts to fill in surveys), if the same account re-started the data collection several times from different IP addresses, if a participant's browser window size was unusual, and other indicators.

The data were collected in batches. After detecting non-compliant participants in a batch, their ratings were excluded from the counters of the number of ratings per dimension per webpage. The subsequent batch automatically prioritized the collection of ratings for the dimensions and webpages that had received the fewest ratings. This approach minimized the impact of non-compliant participants from previous batches on the overall rating sample.

## Limitations

The dataset may have several limitations. Some non-compliant crowdworkers likely evaded detection as such, and their data remained in the sample, adding random noise to the average ratings per webpage per dimension. Participants only briefly viewed the stimuli and did not interact with them, which limits the uses of the dataset outside research on webpage first impression and pre-use preference. The participants were predominantly native English speakers, which limits the cross-cultural generalizability of dataset-based findings. The high prevalence of US banks (approximately two thirds of the commercial banking sample) and universities (approximately half of the tertiary education sample) may also limit the generalizability. The ecommerce sample may have limited visual variability, as even the crowdworkers noted that most ecommerce webpages looked visually appealing and professionally designed. Finally, the screenshots were saved as images with lossy compression (as JPEG files) to reduce their file size, which resulted in some information loss and potentially introduced visual artifacts in the images.

## Ethics Statement

The work involved human subjects, whose consent was obtained prior to data collection. The Board for Ethical Questions in Science of the University of Innsbruck reviewed and approved the project that the data collection was a part of (ethics protocol number *03/2020*).

## CRediT authorship contribution statement

**Aliaksei Miniukovich:** Conceptualization, Methodology, Software, Investigation, Resources, Data curation, Writing – original draft, Project administration, Funding acquisition. **Kathrin Figl:** Conceptualization, Methodology, Writing – review & editing, Supervision, Project administration, Funding acquisition.

## Data Availability

Web Design Prototypicality (Reference data) (Dataverse).
